# Double Metal Oxide Electron Transport Layers for Colloidal Quantum Dot Light-Emitting Diodes

**DOI:** 10.3390/nano10040726

**Published:** 2020-04-11

**Authors:** Myeongjin Park, Jeongkyun Roh, Jaehoon Lim, Hyunkoo Lee, Donggu Lee

**Affiliations:** 1 Department of Electrical and Computer Engineering, Inter-university Semiconductor Research Center, Seoul National University, Seoul 08826, Korea; lightjin@snu.ac.kr; 2 Department of Electrical Engineering, Pusan National University, Busan 46241, Korea; jkroh@pusan.ac.kr; 3 Department of Energy Science, Center for Artificial Atoms, Sungkyunkwan University (SKKU), Suwon, Gyeonggi-do 16419, Korea; j.lim@skku.edu; 4 Department of Electronics Engineering, Sookmyung Women’s University, Seoul 04310, Korea; lhk108@sookmyung.ac.kr; 5 Realistic Media Research Center, Innovative Technology Research Division, Gumi Electronics & Information Technology Research Institute (GERI), Gumi, Gyeonsangbuk-do 39253, Korea

**Keywords:** quantum dot (QD), light emitting diode (LED), metal oxide, double electron transport layer (ETL), SnO_2_ nanoparticles

## Abstract

The performance of colloidal quantum dot light-emitting diodes (QD-LEDs) have been rapidly improved since metal oxide semiconductors were adopted for an electron transport layer (ETL). Among metal oxide semiconductors, zinc oxide (ZnO) has been the most generally employed for the ETL because of its excellent electron transport and injection properties. However, the ZnO ETL often yields charge imbalance in QD-LEDs, which results in undesirable device performance. Here, to address this issue, we introduce double metal oxide ETLs comprising ZnO and tin dioxide (SnO_2_) bilayer stacks. The employment of SnO_2_ for the second ETL significantly improves charge balance in the QD-LEDs by preventing spontaneous electron injection from the ZnO ETL and, as a result, we demonstrate 1.6 times higher luminescence efficiency in the QD-LEDs. This result suggests that the proposed double metal oxide ETLs can be a versatile platform for QD-based optoelectronic devices.

## 1. Introduction

For a few decades, there has been a growing interest in exploiting unique and superior optical properties of colloidal quantum dots (QDs) in various optoelectronic devices [1,2,3,4]. Near-unity photoluminescence quantum yield (PL QY), narrow emission spectral bandwidth with the full width at half maximum around 30 nm, and easily tunable emission wavelength are the attractive features of QDs for display [5,6,7]. Furthermore, low-cost solution processability and flexibility are also appealing properties of QDs to enable next-generation displays. Based on these advantages, the various QDs comprised of II-VI [8,9], III-V [10,11,12], IV [13,14], perovskite [15,16], are actively developing for display application.

The efficiency of QD-based light-emitting diodes (QD-LEDs) has been improved steadily over the past two decades, and one of the most important breakthroughs was made by employing a metal oxide semiconductor in the electron transport layer (ETL). Suitable energy levels and high electron mobility of metal oxide semiconductors led to efficient charge injection and transport, thereby resulting in notable progress in the luminescence efficiency of QD-LEDs [17,18,19,20]. Various types of metal oxide semiconductors have been employed and investigated, and among those metal oxide semiconductors, zinc oxide (ZnO) has been the most widely employed because of their easy processability, excellent electrical properties, and transparency [21,22]. As a result of intensive investigation, the ZnO ETL is now considered as a standard platform for high-performance QD-LEDs, and the state-of-the-art QD-LEDs also employ ZnO-based ETL [10,22].

Although the performance of QD-LEDs has been developed with the ZnO ETL, ZnO has been often indicated as a major cause for undesirable device performance such as the efficiency droop (i.e., a rapid decrease in the efficiency as current density or luminance increase), poor operational stability, and short lifetime. These features are originated from the non-balanced electron and hole densities in the device, which is attributed to excessive electron injection from the ZnO ETL. It is reported that the similar conduction band edge of ZnO to that of QDs often yields spontaneous electron injection, and faster electron mobility of ZnO than hole mobility of typical organic hole-transporting layer (HTL) materials further worsen the charge imbalance [23].

In order to address this issue and improve the charge balance inside the device, various studies have been employed to suppress electron injection from ZnO. One of the most representative approaches is to employ an interlayer between the ZnO ETL and the QD layer [22,24,25,26,27]. Inserting an ultrathin dielectric has been found to be an efficient way to improve the charge balance by reducing the injection rate of electrons and preventing spontaneous injection from ZnO. Diverse organic insulating materials such as poly(methylmethacrylate) (PMMA) [22] and poly(4-vinylpyridine) (PVPy) [24] have been employed and shown their effectiveness. However, employing an insulating interlayer accompanies a careful optimization of the interlayer thickness because the carrier injection through an insulator relies on a direct or Fowler–Nordheim tunneling. Therefore, with the interlayer thicker than the critical thickness, a carrier injection efficiency decreases significantly, resulting in rapid deterioration of device performance.

An alternative approach is to use an n-type organic semiconductor such as poly(9-vinlycarbazole) (PVK) [25], 1,3,5-tris(2-N-phenylbenzimidazolyl)benzene (TPBi) [26], and 4,7-diphenyl-1,10-phenanthroline (Bphen) [27] for the interlayer. In this case, the organic interlayer acts as a second ETL that slows down the injected electrons from ZnO before they reach the QDs. This ‘double ETL’ approach successfully improved the charge balance, but employing organic semiconductors has a drawback in limited processability due to the non-robustness of organic thin films. Therefore, it is necessary to consider other types of materials to construct the double ETL that can efficiently redeem the ZnO-based single ETL while providing unconstrained processability.

In this study, we introduce a double ETL consisting of two types of metal oxide semiconductor to demonstrate the improved performance of QD-LEDs. The double ETL comprises bilayer stacks of ZnO and tin dioxide (SnO_2_) nanoparticles (NPs), and here, SnO_2_ NPs are used to supplement the ZnO ETL; the charge imbalance caused by excessive electron injection from ZnO is controlled by the SnO_2_ second ETL. Employing the material for the second ETL from the library of metal oxide semiconductors has an advantage in processability over organic semiconductors; similar to ZnO NPs, SnO_2_ NPs produce highly smooth and robust thin films under low temperature. As a result of improved charge balance using the ZnO/SnO_2_ double ETL, the QD-LED exhibits notable improvement in luminescence efficiency, suggesting that the double metal oxide ETL could be a new platform to achieve high-performance QD-LEDs.

## 2. Experiments

The SnO_2_ NPs with a diameter of around 5 nm, dispersed in water, was purchased by MKnano (Missisauga, Canada). The details on the material preparation of ZnO NPs with a diameter of 3−5 nm and CdSe/ZnS QDs with a diameter of 8 nm for red emission are followed by our previous articles [21]. For thermal evaporation, 4,4′,4′′-Tris(carbazol-9-yl)triphenylamine (TCTA) and MoO_3_, were purchased at Lumtec (New Taipei City, Taiwan) and Al were purchased at iTASCO (Seoul, Republic of Korea). All materials were used as received.

**Figure 1:**
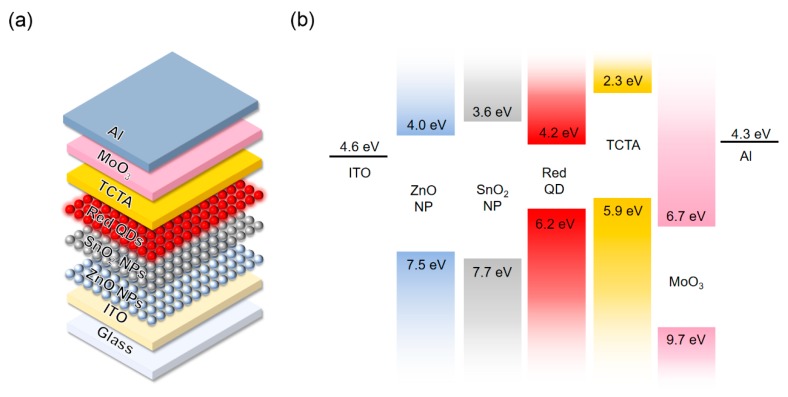
(**a**) Device structure and (**b**) energy band diagram of quantum dot light-emitting diodes (QD-LEDs).

QD-LEDs with the structure of glass/indium tin oxide (ITO)/ETLs (40 nm)/QDs (20 nm)/TCTA (40 nm)/MoO_3_ (10 nm)/Al (100 nm) were fabricated as following sequence (Figure 1a). The ITO glass substrates were sequentially ultrasonicated with acetone, isopropyl alcohol, and deionized (DI) water and then, dried at 120 °C in an oven for 30 min. For single-layer ETL deposition, 40 nm of ZnO NPs thin films were deposited on the cleaned ITO glass by the spin coating method with 20 mg/mL of ZnO NPs solution dispersed in butanol at 2000 rpm during 60 s. For double-layer ETL deposition, first, 10 mg/mL of ZnO NPs solution dispersed in butanol was spin-coated on the cleaned ITO glass at 2000 rpm during 60 s. The thickness of ZnO NPs for the first ETL was 20 nm. Then, 7.5 mg/mL of SnO_2_ NPs solution dispersed in DI water was spin-coated on the ZnO NPs film at 2000 rpm during 60 s. The total thickness of the double ETLs was 40 nm. After each spin coating process to deposit ETL, the processed films were dried for 30 min in a 90 °C nitrogen oven. Then, 20 nm thickness of the QD emission layer was deposited on ETLs by spin-coating at 4000 rpm during 30 s, followed by drying in the 70 °C nitrogen oven for 30 min. Then, substrates were moved to the vacuum chamber, and 40 nm thickness of TCTA, 10 nm thickness of MoO_3_ and 100 nm thickness Al were successively deposited by thermal evaporation method at a pressure below 5 × 10^⁻6^ Torr. After the deposition, all devices were glass capped with ultraviolet (UV) curing epoxy resin (XNR 5570-B1, Nagase ChemteX Corp., Osaka, Japan) in a nitrogen glove box.

The surface topography and Kelvin probe force microscopy (KPFM) of QDs and NPs were obtained using an atomic force microscope (AFM, XE-100, Park Systems, Suwon, Republic of Korea). The work function of QDs was calculated with calibrating the scanning probe tip with a highly oriented pyrolytic graphite (HOPG). The thickness of thin-film was measured by ellipsometry (MG-1000, Nanoview, Ansan, Republic of Korea). QD-LED performance was characterized by a Keithley 236 source measurement unit and CS-1000A spectroradiometer (Konica-Minolta, Tokyo, Japan).

## 3. Results and Discussion

We chose SnO_2_ NPs for the second electron injection layer because of their excellent solution processability, high transparency in the visible region (Figure S1), which is similar to ZnO NPs, and decent electron-transport properties as evidenced by the previous employment in perovskite solar cells [28] and organic light-emitting diodes [29]. In addition, in our recent study [20], we have examined the use of SnO_2_ NPs for the ETL in QD-LEDs, and found out SnO_2_ NPs have favorable interfacial properties with QDs that do not induce spontaneous electron injection; SnO_2_ NPs have lower conduction band minimum (CBM) (see Figure 1b) and lower carrier concentration compared to ZnO NPs. In order to take advantage of SnO_2_ NPs while maintaining an excellent electron transporting ability of the standard ZnO-based ETL, we propose a new ETL platform comprising the stack of ZnO and SnO_2_ NPs. In this double ETL structure, ZnO NPs are responsible for efficient charge transport, and SnO_2_ NPs are responsible for balanced electron-hole densities in the QD-LEDs.

Before employing the double metal oxide ETL in the QD-LEDs, we first examined the morphology of the ZnO/SnO_2_ bilayer stack using atomic force microscope (AFM) measurements and compared them with that of the ZnO film. As shown in Figure S2a,b, the SnO_2_ coated on the ZnO produce a highly dense thin film with the root-mean-square (RMS) roughness of 6.22 nm which is comparable to the RMS roughness of ZnO film, 4.15 nm. Next, we verified the processability of the ZnO/SnO_2_ double ETL in terms of robustness and wettability. In the inverted structure, QDs are deposited on the ETL. Thus, it is necessary to have a robust film that is not damaged during QD deposition. Furthermore, having a wettable surface that does not induce dewetting of QD solution is also important to produce smooth thin film. To check these, we deposited QDs on SnO_2_ film and examined the surface morphology. As a reference sample, we also prepared the QD film coated on ZnO. As shown in Figure S2c,d, as a result of suitable wettability for QD deposition and robustness of SnO_2_ film, the QDs coated on the SnO_2_ film form a highly smooth thin film with a low RMS roughness of 2.05 nm, which is similar to the QD film on ZnO (4.33 nm).

**Figure 2:**
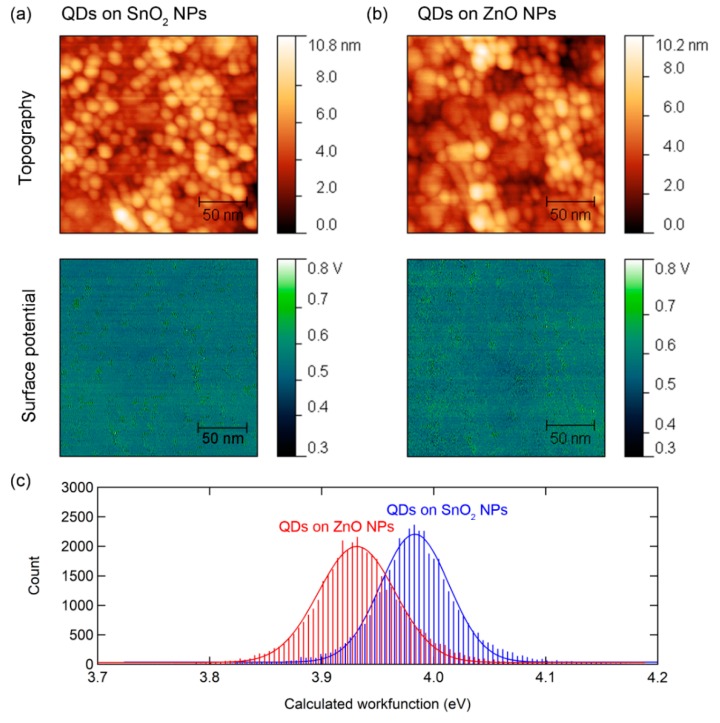
Atomic force microscopy (AFM) topology and surface potential of red QDs on (**a**) SnO_2_ nanoparticles (NPs) and (**b**) ZnO NPs. (**c**) Calculated histogram of the work function of QDs from the surface potential signal with various metal-oxide underlayer. Solid lines denote fitting results with Gaussian distribution.

To further study the electrical interaction between the metal-oxide and QDs, we measured the KPFM of the QDs on SnO_2_ and ZnO. KPFM measure the potential offset between a probe tip and a surface by applying alternating current (AC) voltage, which oscillates the AFM probe tip, and variable direct current (DC) voltage to cancel the oscillations, which are equal to the contact potential difference (CPD) between the probe and sample. The potential difference between the probe tip and sample can be expressed in a difference of work function (W) as follows: *eV_CPD_ = W_sample_ − W_tip_*, where *e* is the elementary charge. Therefore, we can obtain the work function of samples by calibrating the probe tip with HOPG, which has a work function of 4.475eV as a reference surface [30,31,32]. Figure 2a,b showed the topography and surface potential image of QDs on SnO_2_ and ZnO. The uniform surface potential images are due to the evenly spread QD films without pinholes. Figure 2c shows the calculated work function of QDs with different metal oxide underlayer. The histogram of work function was fitted with the Gaussian distribution as follows:

(1) $$f\left(x\right)=Aexp\left[{\left(\frac{x-x_{0}}{width}\right)}^{2}\right]$$

As shown in Figure 2c, the QDs on SnO_2_ had 3.98 eV of center value (*x_0_*) and 0.043 eV of width, and the QDs on ZnO had an average of 3.93 eV of *x_0_* and 0.048 eV of width. A work function is the energy of the Fermi level versus the vacuum level; thus, the low work function of QDs on ZnO can be interpreted as the Fermi level rising due to spontaneous electron injection from ZnO to QDs. In this regard, the high work function of QDs on SnO_2_ is an indication of reduced spontaneous electron injection from the underlying SnO_2_ layer to QDs. Therefore, it can be expected that the metal oxide ETL will influence on the device performance due to the difference in electrical interaction with QD.

**Figure 3:**
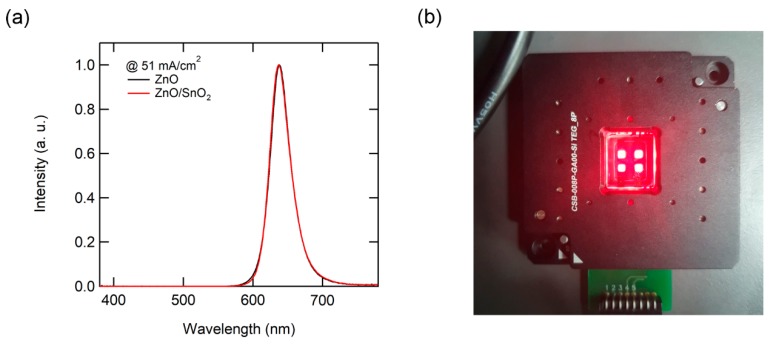
(**a**) Electroluminescence (EL) spectra of QD-LEDs with ZnO and SnO_2_/ZnO double electron transport layer (ETL), and (**b**) operating device image of QD-LEDs with SnO_2_/ZnO double ETL.

Next, we fabricated QD-LEDs based on the ZnO/SnO_2_ double ETL. Figure 3a shows the electroluminescence (EL) spectra of QD-LEDs. The EL spectra of QD-LEDs with the ZnO ETL and the ZnO/SnO_2_ double ETL showed almost identical spectrum shapes, due to the similar optical properties of ZnO and SnO_2_ (i.e., transparency in the visible range). In addition, both QD-LEDs exhibit pure emission of QDs without any parasitic emission from adjacent SnO_2_ and TCTA layers. This indicates that electron and hole are injected to QDs without being accumulated at the ETL and HTL interfaces, and the exciton recombination region is well confined in the QD layer. Figure 3b shows uniform emission of 2 × 2 arrays of QD-LEDs over the glass size of 20 × 20 mm^2^.

**Figure 4:**
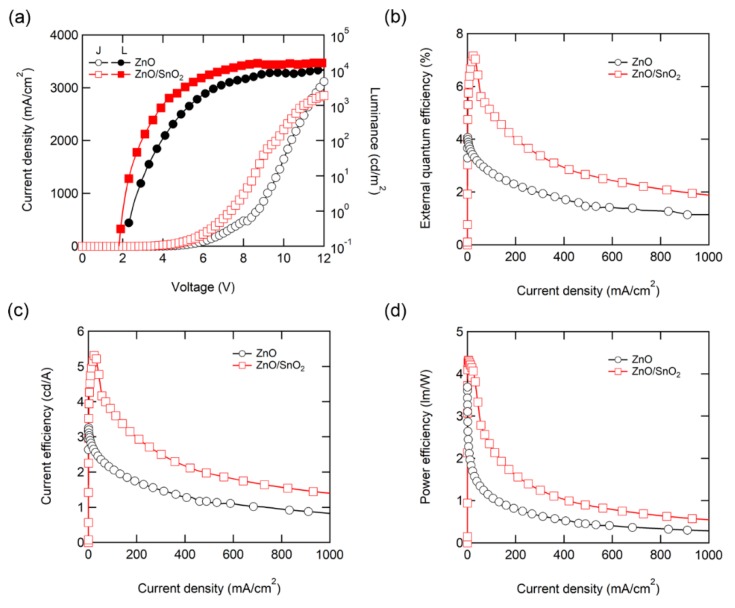
(**a**) Current density (J)-voltage (V)-luminance (L), (**b**) external quantum efficiency (EQE)-J (**c**) current efficiency (CE)-J and (**d**) power efficiency (PE)-J curves of QD-LEDs with ZnO and SnO_2_/ZnO double ETL.

Figure 4 and Table 1 compares QD-LED performances with the single ZnO ETL and ZnO/SnO_2_ double ETLs. Figure 4a shows the current density–luminance–voltage relationship of the QD-LEDs with the ZnO or SnO_2_/ZnO ETLs. As shown in Figure 4a, the QD-LED with the double ETLs exhibits much better switching behaviors with a lower turn-on voltage and steeper increase in both current density and luminance. The QD-LED with the double ETL also exhibited decreased operating voltage. Note that decrease in operating voltage has never been observed in the previous studies that employed a secondary injection layer to suppress spontaneous electron injection from ZnO [22,24]; Indeed, employing the secondary injection layer often result in an increased operating voltage because of poor electrical properties of the secondary injection layer. This distinguishable result shows the superiority of the proposed ETL platform. All efficiency parameters including external quantum efficiency (EQE, Figure 4b), current efficiency (CE, Figure 4c), and power efficiency (PE, Figure 4d) are greatly improved in the overall current density region with the double ETLs. In particular, the peak EQE of the QD-LEDs was notably increased from 4.11% to 7.16% by using the double ETLs. In the typical CdSe QD-LEDs with an inverted device structure based on the ZnO ETL, charge imbalance is occurred due to the spontaneous electron injection from ZnO, thereby resulting in efficiency deterioration [23]. Therefore, this enhancement in EQE is attributed to the improved electron and hole balance in the QD emission layer by the employment of the SnO_2_ second ETL. As a result of improved charge balance inside the QD-LEDs, improved current efficiency and power efficiency were also obtained.

In the luminance (L)-voltage (V) characteristic, luminance saturates as the driving voltage increases, which is interpreted as an increase of exciton quenching at a high charge carrier density, resulting in efficiency roll-off. Although the efficiency of QD-LEDs has increased with double ETL, efficiency roll-off still remained. This is caused by the characteristics of thin EMLs, which is comprised of around two monolayers. Further optimization of the EML layer is needed to improve efficiency roll-off, such as increasing the shell thickness of QDs [33] or increasing the thickness of EML through the hybridization of QDs with polymer matrix [34].

## 4. Conclusions

To summarize, we have introduced double metal oxide ETLs as a new platform to achieve high-performance QD-LEDs. The proposed ETL platform comprises stacks of the primary ZnO ETL and the second ETL comprises SnO_2_ NPs. The second SnO_2_ ETL is employed to supplement the standard ZnO ETL in suppressing spontaneous electron injection from ZnO to QDs. With the SnO_2_ second ETL, charge balance in the QD-LEDs is improved, thereby exhibiting a significant increase in electroluminescence efficiency. The excellent processability and robustness of the proposed double metal oxide ETLs exhibit promising potential as the new platform for QD-based optoelectronic devices.

## Figures and Tables

**Table 1:** QD-LED performances with with ZnO and SnO_2_/ZnO double ETL.

Parameter	ZnO NPs (40 nm)	SnO_2_ NPs/ZnO NPs (20 nm)/(20 nm)
J (mA/cm^2^) @ 5 V	33.7	80.44
L (cd/m^2^) @ 5 V	848	3061
CE_max_ (cd/A)	3.2	5.31
EQE_max_ (%)	4.11	7.16
PE_max_ (lm/W)	3.86	4.32

## Supplementary Materials

The following are available online at https://www.mdpi.com/2079-4991/10/4/726/s1: Figure S1: Transmittance spectra of ITO, ZnO NPs and SnO_2_ NPs.; Figure S2: AFM topology of (a) ZnO NPs, (b) SnO_2_ NPs on ZnO NPs, (c) QDs on ZnO NPs and (d) QDs on SnO_2_ NPs.
