# Supplementary material for: Double Metal Oxide Electron Transport Layers for Colloidal Quantum Dot Light-Emitting Diodes
Source: Nanomaterials (Basel). 2020 Apr 11;10(4):726. doi: 10.3390/nano10040726 (PMC7221608; doi:10.3390/nano10040726)
Supplement: Supplementary file 1 [file nanomaterials-10-00726-s001.pdf]

nanomaterials

Supplementary Materials

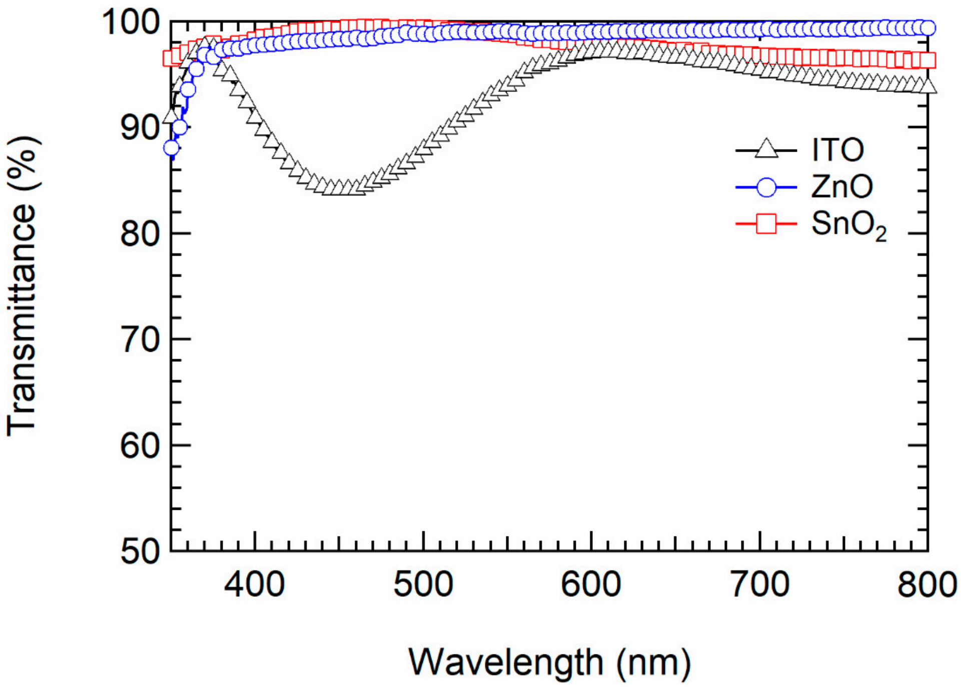

Figure S1. Transmittance spectra of ITO, ZnO NPs and SnO_2 NPs.

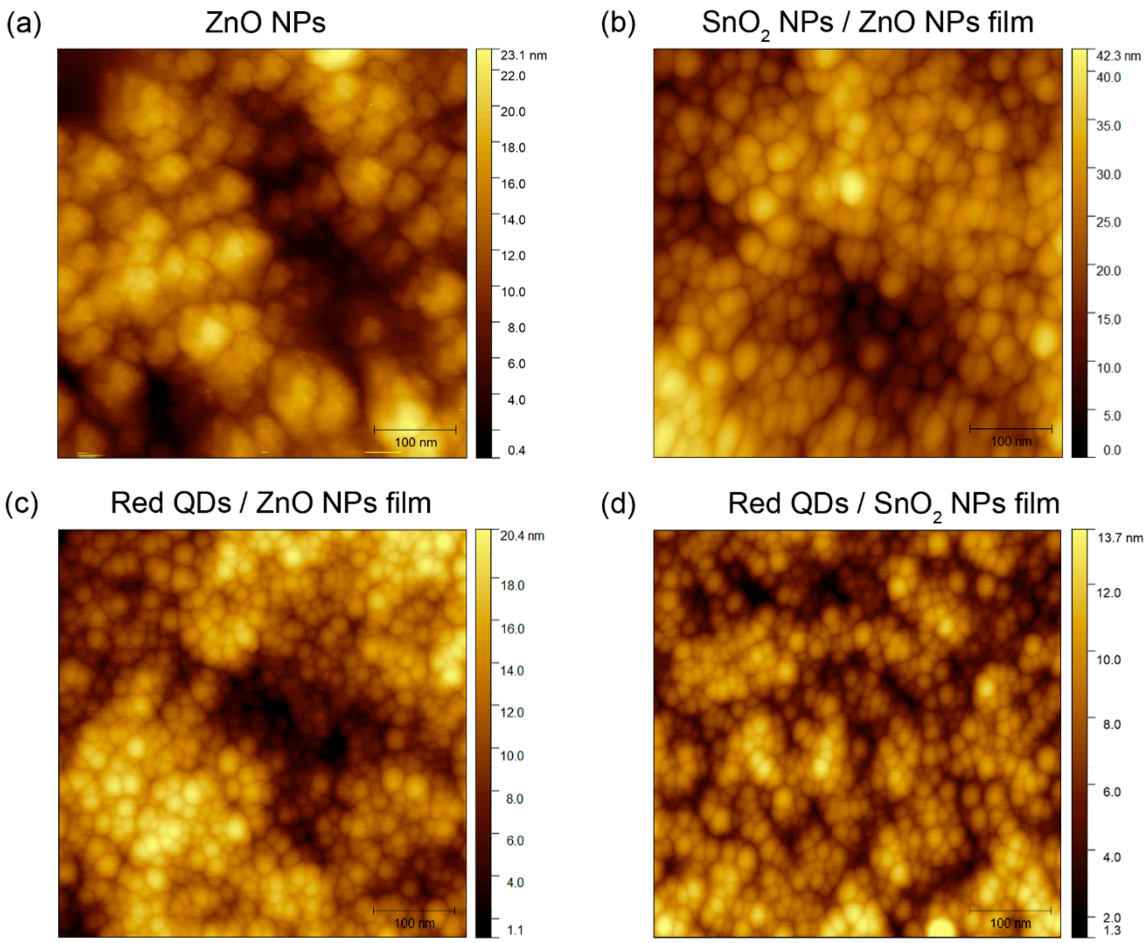

Figure S2. AFM topology of (a) ZnO NPs, (b) SnO_2 NPs on ZnO NPs, (c) QDs on ZnO NPs and (d) QDs on SnO_2 NPs.

© 2020 by the authors. Licensee MDPI, Basel, Switzerland. This article is an open access article distributed under the terms and conditions of the Creative Commons Attribution (CC BY) license (http://creativecommons.org/licenses/by/4.0/).
